# A lightweight intelligent model for VOC mixture analysis: toward preclinical breath biomarker analysis

**DOI:** 10.3389/fbioe.2026.1821312

**Published:** 2026-06-03

**Authors:** Xinzhe Fang, Chengyuan Zha, Weihua Fang

**Affiliations:** 1 School of Pharmacy, Macau University of Science and Technology, Macau, China; 2 School of Artificial Intelligence and Automation, Hohai University, Nanjing, China; 3 Nanjing Research Institute of Water Conservancy and Hydrology Automation, Ministry of Water Resources, Nanjing, China

**Keywords:** cross-channel global correlation, electronic nose, lung cancer, manual feature engineering, temporal evolution characteristics

## Abstract

For the current noninvasive lung cancer screening methods based on volatile organic compounds (VOCs) using electronic noses (e-noses), existing approaches still face limitations in modeling the long-range dependencies of sensor responses, the cross-channel global correlations, and the long-term trend features during the steady-state phase. Moreover, the associated deep learning models are often structurally complex and rely heavily on manual feature engineering, which restricts the engineering application and clinical translation of e-nose systems. To address these issues, this study proposes a lightweight global–local feature fusion framework for complex VOC sensing tasks and designs an efficient, lightweight feature extraction module (LFE) to achieve high-efficiency gas classification. For quantitative analysis of gas components, a GBDT–GRU Joint Prediction Model (JGPM) is introduced, effectively modeling the temporal evolution characteristics of sensor response signals. The above models were systematically validated using an e-nose experimental platform with synthetic gases of acetone, ethanol, isopropanol, and their mixtures at the ppm level as a proof-of-concept (PoC) study. The experimental results show that the proposed models outperform the comparative methods in both gas classification accuracy and concentration prediction performance, while maintaining low model complexity. Although current validation is at the preclinical stage, this framework provides a robust algorithmic foundation for future intelligent gas sensing and clinical breath-based disease screening.

## 1 Introduction

Lung cancer (LC) has a 5-year survival rate of only 19.7%, and its high incidence and lethality pose a significant threat to human health and life (Huang et al., 2020; Vranas et al., 2020). However, the 5-year survival rate for early-stage lung cancer can exceed 80% (Liang et al., 2023), making early screening crucial for improving patient survival. Studies have shown that lung cancer can lead to elevated concentrations of acetone, ethanol, and 2-propanol in exhaled breath, which have been identified as potential early biomarkers for lung cancer (Buszewski et al., 2012; Ulanowska et al., 2011). The concentration range of acetone in the exhaled breath of healthy individuals is 41.6–753.4 ppb, the concentration range of ethanol is 4.5–479.5 ppb, and the median concentration of 2-propanol is 13.3 ppb. In contrast, the concentration range of acetone in the exhaled breath of lung cancer patients is 112.3–2653.7 ppb, ethanol ranges from 12.8 to 1520.1 ppb, and the concentration of 2-propanol ranges from 8.7 to 989.2 ppb. Therefore, achieving high-precision identification of these three gases holds significant diagnostic value.

Gas identification methods include gas chromatography–mass spectrometry (GC–MS) (Einoch Amor et al., 2023), spectroscopic detection (Li S. et al., 2023), and e-nose detection (Li et al., 2025a). Compared with gas chromatography–mass spectrometry and spectroscopic detection, sensor array–based e-nose systems have become one of the key technologies for gas detection and identification due to their simple structure and strong scalability. E-noses analyze the output signals of sensor arrays using pattern recognition algorithms, offering advantages such as low cost, wide applicability, and real-time monitoring capabilities. However, e-noses face the issue of cross-sensitivity, meaning they may produce similar responses to different gases. To address this, researchers typically employ advanced feature extraction techniques–such as Principal Component Analysis (PCA) and Independent Component Analysis (ICA) (Zhu et al., 2022) –or feature selection algorithms to remove redundant data. Subsequently, they apply advanced pattern recognition algorithms, like Support Vector Machines (SVM), Random Forests, or neural networks, to process complex feature spaces and accurately identify target gases. Interestingly, rather than viewing cross-sensitivity purely as a drawback, e-nose systems leverage an array of cross-reactive sensors, which obviates the need for targeted quantification of individual VOCs. Instead, these sensors generate holistic response profiles derived from the collective interaction of the gaseous mixture, enabling precise classification through integrated chemometric methods and pattern recognition algorithms (Govindarajan et al., 2025). Recent advancements underscore the exceptional versatility of such cross-reactive sensing platforms; by analyzing aggregate VOC “fingerprints” rather than isolated analytes, this technology can be seamlessly adapted to diverse application scenarios. Serving as a cost-effective, non-invasive, and rapid alternative to conventional analytical methods, it has been successfully deployed in fields ranging from food quality control (e.g., honey adulteration detection and fish freshness assessment) to clinical diagnostics (e.g., non-invasive lung cancer screening), thereby demonstrating the broad applicability and robustness of cross-reactive sensor arrays (Abraham and Binson, 2025; Wu et al., 2023; Chaudhary et al., 2024).

Feature extraction, feature processing, and pattern recognition methods directly affect the detection performance of e-noses. Özsandikcioğlu and Atasoy (2023) proposed a hybrid multi-sensor system aimed at improving the detection performance of VOCs in the breath samples of lung cancer patients. By analyzing breath samples from lung cancer patients and healthy individuals, and combining PCA and LDA for dimensionality reduction with SVM and RF classification models, the system achieved a maximum detection accuracy of 95.36%. Brinkman et al. (2019) proposed a feature extraction method for defining severe asthma phenotypes and assessing their stability. Using a composite e-nose platform to collect patients’ exhaled breath samples, the generated raw feature set was optimized through ComBat batch correction and BoxCox transformation to improve data distribution. After data normalization, principal component analysis (PCA) was applied for dimensionality reduction to retain key features and reduce redundancy. Clustering analysis using K-means then identified three distinct asthma phenotypes. Binson et al. (2024b) extracted respiratory features from exhaled breath using metabolomics approaches, applied kernel principal component analysis (kPCA) for nonlinear dimensionality reduction, and employed the extreme gradient boosting (XGBoost) algorithm for classification. This method effectively differentiates lung cancer stages and pathological types. Liu et al. (2021) proposed an efficient feature extraction workflow combined with a novel E-nose platform for precise lung cancer identification. By extracting 10 traditional composite features and three exponential moving average (EMA) features, they constructed a 247-dimensional high-dimensional feature vector. Using the sparse group lasso (SGL) feature selection method, 19 key features were identified, and classic classifiers were employed to validate the feasibility of lung cancer detection based on VOCs. Previous studies have shown that the feature extraction process relies heavily on domain expert knowledge, and traditional statistical and transform-domain features are insufficient to comprehensively capture the complex spatiotemporal patterns in sensor signals, especially under the nonlinear interaction effects present in multi-gas mixtures. Moreover, manually crafted features are typically optimized for specific experimental environments and sensor arrays, making it difficult to adapt to changes in detection conditions or hardware replacements, which significantly reduces the model’s generalization ability. Processing high-dimensional time-series signals faces the curse of dimensionality, and traditional dimensionality reduction methods may compromise the critical spatiotemporal correlations in the original data. Additionally, shallow models struggle to accurately capture the nonlinear superposition of gas responses. The trial-and-error nature and parameter tuning involved in feature engineering are cumbersome and inefficient, limiting the model’s portability and the practical deployment of the approach.

Convolutional neural networks (CNNs) integrate feature extraction, feature processing, and pattern recognition into a single network framework. Through end-to-end learning, they avoid the complexity and subjectivity associated with traditional feature extraction methods (Zhao et al., 2024). Lee et al. (2024) developed a breath analysis system that integrates multimodal sensor arrays with deep learning, innovatively using a 1D convolutional neural network to directly process sensor time-series signals without manual feature extraction. Through 5-fold cross-validation, the system significantly outperformed single sensor arrays and other deep learning models. Avian et al. (2022) proposed a convolutional neural network architecture combining Kernel Principal Component Analysis (KPCA) and Fx-ConvNet for analyzing VOC signals collected by e-nose systems. Compared with existing machine learning algorithms, the proposed KPCA + Fx-ConvNet model achieved the best performance in both 3-fold and 5-fold cross-validation. Although convolutional neural networks (CNNs) are widely used in gas classification, their large number of trainable parameters, high computational complexity, and relatively slow inference speed limit their portability in embedded systems. To address these issues, Zha et al. (2024) proposed a lightweight neural network, LTNet, for high-precision classification of breath biomarkers in lung cancer patients; however, this approach does not model the global correlations among features. Shi et al. (2023) proposed a gas feature attention mechanism (GFAM) that integrates peak factors, integral values, and steady-state means to capture the temporal and steady-state characteristics of e-nose data; however, the depth of inter-channel information interaction is limited. In the aforementioned models, convolutional neural networks (CNNs) are typically used as the core feature extractors in deep learning architectures. While CNNs excel at extracting local dynamic features due to their limited receptive fields, they still face certain limitations in modeling long-range dependencies, global correlations, and long-term trend features present during the steady-state phase (Ma et al., 2023; Wang et al., 2019). To more comprehensively capture both spatial and temporal features, recent studies have explored the integration of image transformation techniques with hybrid architectures. Ni et al. (2024) employed Gramian Angular Field (GAF) and Markov Transition Field (MTF) to convert time-series data into images, and developed a multi-task CNN–LSTM network to simultaneously model spatial patterns and temporal dependencies.

Li et al. (2024) proposed a lightweight hybrid network, PSCFormer, which employs LPSF, CE, and TE modules to respectively focus on key gas features, model local dependencies, and capture global correlations. A feature complementarity mechanism is used to fuse local and global information, effectively balancing performance and complexity while alleviating the limited receptive field of CNNs and the high data requirements of Transformers. Similarly, Ni et al. (2025) proposed the Scentformer architecture, which integrates convolutional neural networks (CNNs) with multi-head attention mechanisms. Through SHAP analysis, they demonstrated that this hybrid design effectively compensates for CNNs’ limitations in modeling global temporal features; moreover, by incorporating transfer learning, the model achieves significantly improved generalization in large-scale odor recognition tasks. Although the aforementioned methods have achieved some success in modeling local and global features, their feature fusion strategies remain relatively simplified, leaving room for further optimization in modeling the importance of different features and enabling cross-layer feature interactions. Furthermore, while recent advancements have introduced multitask deep learning frameworks to achieve simultaneous qualitative classification and real-time concentration prediction for individual VOCs (Ni et al., 2025), current studies still predominantly focus on individual VOC biomarkers or pattern classification based on limited VOC combinations. Systematic quantitative analysis and decoupling of the concentrations of each component in complex mixed gases, however, remains largely unexplored (Fan et al., 2024). This lack of quantitative studies on mixed breath biomarkers in lung cancer patients has become a key factor limiting the clinical translation of breath-based diagnostic technologies. Given the extreme complexity and uncontrollable nature of clinical breathomics at the parts-per-billion (ppb) level, conducting proof-of-concept studies with ppm-level synthetic gas mixtures—in line with recent cutting-edge paradigms—is an indispensable foundational step. This ensures robust verification of the model’s capacity to extract and decouple the underlying features from complex sensory data. Therefore, as a preclinical proof-of-concept study, this work addresses the algorithmic limitations of existing methods for breath biomarker detection. These existing approaches struggle to achieve both high-precision gas classification and multi-component concentration quantification in complex mixed VOC scenarios. Furthermore, they inadequately model temporal dynamics and cross-sensor global information, and face challenges in balancing model complexity with deployability. To overcome these issues, a lightweight Local–Global Feature Fusion Network (LLGFN) and a GBDT–GRU Joint Prediction Model (JGPM) are proposed. The organization and main content of this paper are as follows. To reduce the complexity of manual feature extraction, Chapter 2 reconstructs multi-sensor time-series data into GASF images and discusses the effects of different reconstruction scales on feature discriminability and computational cost. Next, to address the redundancy of information and the need for multi-scale features in lung cancer gas recognition—while maintaining high feature representation capability and significantly reducing model parameters—Chapter 3 constructs a lightweight classification network that fuses local features (via cross-stage feature connections) and global features (using a downsampled Transformer encoder reconstructed with depthwise convolutions). To address issues of computational redundancy, parameter overload, and gradient vanishing in neural networks on resource-constrained devices, while maintaining high feature representation capability, a lightweight feature extraction module (LFE) tailored for intelligent sensing scenarios was designed.

Furthermore, to address the challenge of effectively modeling the temporal evolution of sensor response signals in gas concentration prediction, Chapter 3 proposes a GBDT–GRU Joint Prediction Model (JGPM). Finally, the paper presents the research conclusions and outlines directions for future work.

## 2 Experimental design and data preprocessing

### 2.1 Dataset collection

**FIGURE 1:**
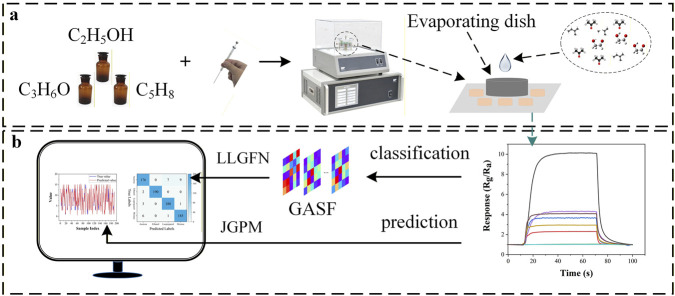
Schematic diagram of the overall process of this work: **(a)** experimental process of the gas sensing system; **(b)** pattern recognition process.

This study utilized the CGS-8 intelligent gas sensing analysis system provided by Beijing Elitech Technology Co., Ltd. The entire experimental process is shown in Figure 1a, where the sensor array consists of eight types of sensors, with two sensors of each type, totaling 16 sensors. The detailed information for each sensor is provided in Table 1. Figure 1b illustrates the pattern recognition process.

**TABLE 1:** Sensor models and optimal operating currents.

NO.	Models	Target gases	Detection ranges (ppm)	Optimal operating currents (mA)
1	TGS2600	Ethanol, hydrogen	1–30	45
2	WSP2110	​	1–50	42
3	TGS2610	Organic compounds	500–10000	55
4	TGS822	​	Ethanol 50–500	120
5	TGS2620	Ethanol, organic compounds	Ethanol 50–5,000	43
6	MQ135	​	Ethanol 10–300	125
7	MQ3	Ethanol	Ethanol 25–500	130
8	TGS2602	Ammonia, ethanol	Ethanol 1–30	50

### 2.2 Experimental design

The experiment selected acetone, ethanol, and isopropanol as three representative VOCs, along with their equimolar multi-component mixtures, as the target analytes. Gas samples were prepared using the static mixing method with stock solutions of acetone (98%), ethanol (98%), and isopropanol (99.7%). The concentrations of both single-component gases and mixed gases were set to 1, 3, 5, 7, 9, 11, 13, and 15 ppm, while the mixed gases were prepared by combining the three VOCs at equal concentrations. To account for the significant influence of temperature and humidity on sensor responses (Binson et al., 2021), all experiments were conducted under controlled environmental conditions at 25 °C ± 3 °C and 50% ± 10% RH. Furthermore, the sensor array was preheated for 1 h prior to each measurement to ensure repeatability and minimize sensor drift (Binson et al., 2024b). For each gas exposure cycle, both the response time and recovery time were set to 120 s, resulting in a total of 1920 sampling points for each experimental group.

The liquid to be tested was precisely extracted using a micro-injector with a range of 10 µL. According to Equation 1, directly using the high-concentration stock solution would result in too small an extracted volume, making it difficult to complete the injection into the gas chamber. To ensure an adequate gas volume and controllable concentration, the stock solution was diluted to 10% before preparation for the experiment.

$$Q=\frac{V\times C\times M}{22.4\times d\times r}\times 10^{-9}\times \frac{273+T_{R}}{273+T_{B}}$$ (1)

In the equation, *Q* and *V* represent the volume of the liquid to be tested and the gas chamber volume (mL), *C* is the desired gas concentration (ppm), *M* is the molecular weight of the substance ( g/mol ), d is the concentration of the liquid to be tested (%), *r* is the liquid density ( $g/{\text{cm}}^{3}$ ), and $T_{R}$ and $T_{B}$ are the ambient temperature and gas chamber temperature (°C), respectively.

### 2.3 Data preprocessing

**FIGURE 2:**
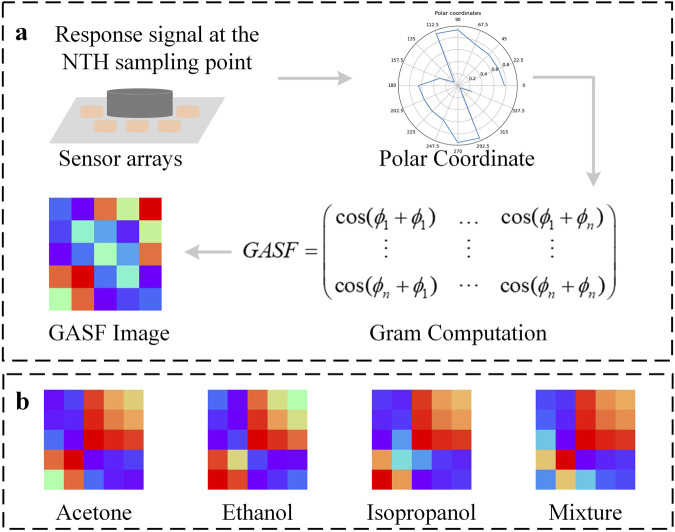
**(a)** GASF image encoding process, **(b)** encoded image.

Traditional time-series data processing methods often suffer from limitations such as subjectivity and decoupling from pattern recognition, which may result in the loss or distortion of important features. Transforming raw signals into image representations that can serve as effective inputs to CNNs enables better capture of the spatial features of the same gas across different sensors while preserving temporal dependencies, allowing the model to learn inter-sensor relationships. Compared with traditional time-series analysis, the Gramian Angular Summation Field (GASF) generates images by computing the cosine summation of angular values between multiple data points (Zhu et al., 2024; Xiong et al., 2023). By aggregating multiple raw signals, the influence of local noise on the results is ultimately averaged out. The smoothed image data enable the model to focus on important features without being disturbed by local random fluctuations, thereby reducing sensitivity to noise. Meanwhile, image representations can more comprehensively integrate spatial and temporal information, and this rich form of representation enables the model to extract more complex features, thereby improving model robustness. Figure 2 illustrates the transformation process of encoding raw response data into two-dimensional color images. After mapping the response data of the sensor array at the Nth sampling point into the polar coordinate system, the cosine summation of angular values between each pair of responses is calculated by constructing a Gramian matrix, which is then reconstructed into a 5 × 5 two-dimensional matrix. Subsequently, the matrix is converted into a color image in JPG format through color mapping, with a size of 470 × 470, a resolution of 100 DPI, and a bit depth of 24 bits. This encoding strategy not only effectively preserves the spatial and temporal features of the same gas across different sensors and reduces sensitivity to noise, but also overcomes the challenges of manual feature engineering, thereby lowering the risk of data bias and loss.

The reconstruction scale directly determines the spatial resolution and effective capacity of the Gramian representation. A smaller scale introduces stronger information compression, which may result in the loss of key inter-sensor correlation structures. On the other hand, a larger scale retains finer-grained spatial variations but significantly increases feature dimensions and computational overhead. This chapter discusses in detail the classification performance and training efficiency at different reconstruction scales ( k=2∼16 ). The experiments were conducted using the LLGFN network, with consistent training configurations throughout.

**FIGURE 3:**
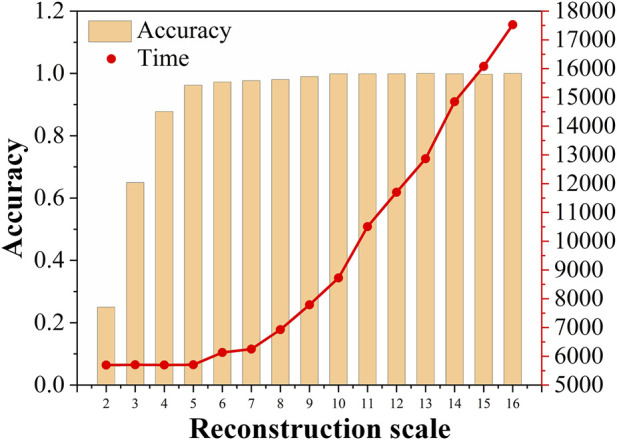
Relationship between reconstruction scale, classification accuracy, and com-putational cost.

The results in Figure 3 indicate a clear trade-off between feature discriminability and computational cost with respect to the reconstruction scale. When k<5 , the reconstructed images fail to adequately capture the correlation structures between sensors, leading to a significant drop in classification accuracy. When 6<k≤16 , the training time increases significantly, while the performance improvement is limited, showing a clear diminishing marginal return characteristic. Considering both classification performance and training efficiency, k=5 lies at the inflection point of the accuracy-efficiency curve, achieving a balanced compromise between the two. Therefore, a 5×5 Gramian reconstruction scale is uniformly adopted in subsequent experiments.

## 3 Model construction

### 3.1 Lightweight framework

**FIGURE 4:**
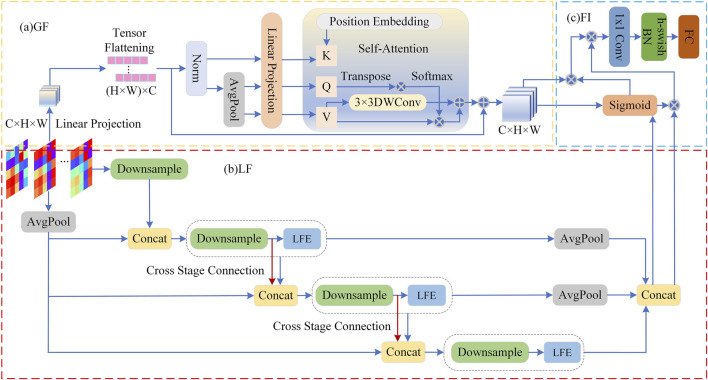
LLGFN network architecture: **(a)** GF denotes the global feature extraction module; **(b)** LF represents the local feature extraction module; and **(c)** FI corresponds to the feature interaction module.

In image processing tasks, the combination of global and local features can enhance the model’s recognition capability. This paper proposes a lightweight neural network, LLGFN, to address the limitations of traditional convolutional neural networks in feature extraction. As shown in Figure 4, LLGFN consists of two core modules: LF (Local Features) and GF (Global Features), which are responsible for extracting local and global features, respectively. The LF module has a dual-branch architecture. The input image with dimensions (H × W × 3) is downsampled through a 3 × 3 convolutional layer, followed by two 3 × 3 convolutions with a stride of 1 for local feature extraction. The resulting feature map is then concatenated with another branch, which has undergone max pooling, to reduce the spatial information loss during downsampling. LFE is primarily responsible for global feature extraction. The LF module enhances the model’s feature representation ability by connecting features across multiple stages, allowing the model to progressively accumulate global information while maintaining local details. This is achieved without increasing the network depth, by expanding the number of channels and increasing the network’s receptive field. The three outputs of the LF module are concatenated and then fed into the feature fusion module. GF is composed of a downsampled Transformer encoder reconstructed through deep convolution, aimed at enhancing the local and global interactions of the input features. Ultimately, the outputs of the LF module and the GF module are fused, enabling high-precision classification of the gas data converted from GASF.

#### 3.1.1 LFE module

The feature maps exhibit high similarity across different channels, and this redundancy leads to increased computational and memory access costs (Liang et al., 2020; Li J. et al., 2023). MobileNetV3 employs a bottleneck structure at specific layers to achieve feature transformation with relatively small input channels and larger internal channels (Howard et al., 2019). This paper designs a lightweight and efficient feature extraction module (LFE) aimed at performing efficient feature extraction while reducing redundant computations and lowering the network’s parameters.

**FIGURE 5:**
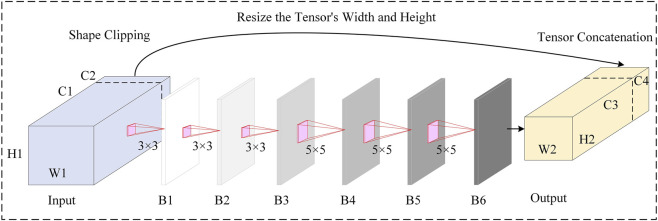
LFE structure diagram, where the input tensor size is H1 x W1 x (C1 + C2), B1–B6 represent six different bottlenecks, and the output tensor size is H2 x W2 x (C3 + C4).

LFE is a lightweight feature extraction module designed for intelligent sensing scenarios. It addresses the three major challenges of deep neural networks on resource-constrained devices: computational redundancy, parameter overload, and gradient vanishing. While maintaining high feature representation capability, it optimally minimizes computational complexity and memory access costs. The overall process is shown in Figure 5. The input tensor is first split along the channel dimension into two paths: one for processing and one for directly passing the raw information through the identity path. The processing path captures features at different levels using multi-scale deep convolutions, with shallow 3 × 3 and deeper 5 × 5 convolutions. These are combined with shallow ReLU and deeper Hard-Swish hybrid activation functions to balance efficiency and expressive power. LFE embeds the SE attention mechanism only at key layers to suppress noisy channels. The features are then concatenated with those from the identity path and undergo channel mixing to enable information interaction. This approach maintains high feature extraction accuracy while reducing model parameters. As the network operates, the identity path tensor, which directly passes raw information, also participates in feature extraction, thereby preventing feature loss. Table 2 provides the specifications of LFE.

**TABLE 2:** LFE specifications.

NO.	Input	Channel expansion	Output	Kernel size	SE	NL	S
B1	Variable	16	16	3	✓	RE	2
B2	16	72	24	3	—	RE	2
B3	24	88	24	3	—	RE	1
B4	24	96	40	5	✓	HS	2
B5	40	240	40	5	✓	HS	1
B6	40	120	Variable	5	✓	HS	1

where SE indicates whether the bottleneck module uses the Squeeze-and-Excite mechanism; NL denotes the type of nonlinear activation function used, with HS representing h-swish and RE representing ReLU; s denotes the stride.

#### 3.1.2 Transformer encoder reconstructed with DW convolution

In gas detection tasks, the sensor array responses typically exhibit significant temporal correlation and coupling characteristics across sensor channels. With its global self-attention mechanism (Si et al., 2022), the Transformer can effectively model the global relationships between different sampling points and sensor channels. However, the computational complexity of the standard self-attention mechanism grows quadratically with the length of the input sequence. This results in high computational and storage overhead in scenarios such as modeling high-resolution gas response images or long-duration time series (Li et al., 2025b), which limits its application in practical gas sensing systems.

To address the above issue, this paper adopts a downsampled Transformer encoder structure based on DW convolution reconstruction. This approach reduces the computational complexity of self-attention while preserving its ability to effectively model key gas features. The images reconstructed based on GASF are divided into several non-overlapping patches and are projected into a high-dimensional embedding space through linear mapping, forming feature tensors.

$$X\in R^{B\times N\times D}$$ (2)

As shown in Equation 2, B represents the batch size, N denotes the sequence length, and D refers to the embedding dimension. To explicitly model the spatial or temporal positional information between patches, position encodings are introduced into the patch embedding sequence.

To reduce the computational complexity of self-attention, layer normalization and average pooling downsampling are applied to the input features before attention computation, as represented by:

$$X_{s}=Pool\left(\mathrm{LN}\left(X\right)\right)$$ (3)

In Equation 3, $X_{s}$ represents the downsampled gas feature tensor. This operation effectively compresses the sequence length, significantly reducing the complexity of subsequent self-attention computations. Building on this, the downsampled features undergo linear mapping to construct the query (Q), key (K), and value (V) matrices. Subsequently, a multi-head self-attention mechanism is used to model the global correlations of the gas response features. The attention weights reflect the degree of correlation between responses from different sampling points and sensor channels, thereby representing the gas response features.

While average pooling downsampling reduces computational complexity, it inevitably introduces local information loss, which in turn affects the ability of the attention output to capture fine-grained response variations. To address this issue, DW Convolution is introduced during the attention output stage to reconstruct the features. DW Convolution performs local feature modeling on a per-channel basis with lower computational overhead. It can compensate for the loss of local response information during downsampling and further refine the value vector features. The final output of the downsampled self-attention module, based on DW reconstruction, can be represented as Equation 4.

$$Y=DWConv\left(Attention\left(Q,K,V\right)\right)$$ (4)

This structure reduces the computational complexity of self-attention while maintaining the ability to model the global correlations of gas response features and represent local detail information. It is suitable for efficient modeling of high-resolution gas images and long-duration gas detection sequences.

#### 3.1.3 Feature interaction

Figure 4c illustrates the feature interaction (FI). In the FI module, the information from the local features (LF) is transformed into weights that influence the global features (GF) through a sigmoid activation function, representing the degree to which LF gas information affects the distribution of GF gas information. The sudden change in local gas responses may lead to significant variations in the global features. Accordingly, the information from the global features (GF) is transformed into weights that influence the local features (LF) through the sigmoid activation function, reflecting the modulation of the local features by the global gas distribution. The FI module enables complementary interactions between local and global features, allowing the final fused features to preserve both local details and overall trends. The specific mathematical process is shown in Equations 5–8:

$$GFWeighted=Sigmoid\left(GF\right)$$ (5)

$$GF^{\prime }=LFWeighted⊙GF$$ (6)

$$LFWeighted=Sigmoid\left(LF\right)$$ (7)

$$LF^{\prime }=GFWeighted⊙GF$$ (8)

Here, GFWeighted represents the weighted influence of local features on global features, $GF^{\prime }$ denotes the global features after adjustment by the local features, ⊙ refers to the element-wise multiplication, LFWeighted represents the weighted influence of global features on local features, and $LF^{\prime }$ denotes the local features after adjustment by the global features.

To effectively fuse local and global features, a comprehensive feature integrating both global and local gas concentration information is constructed. The mathematical formula is expressed in Equation 9:

$$mix\_F=GF^{\prime }+LF^{\prime }$$ (9)

Here, mix_F represents the final output of the comprehensive feature, which is then fed into the fully connected layer.

### 3.2 GBDT-GRU joint prediction model

**FIGURE 6:**
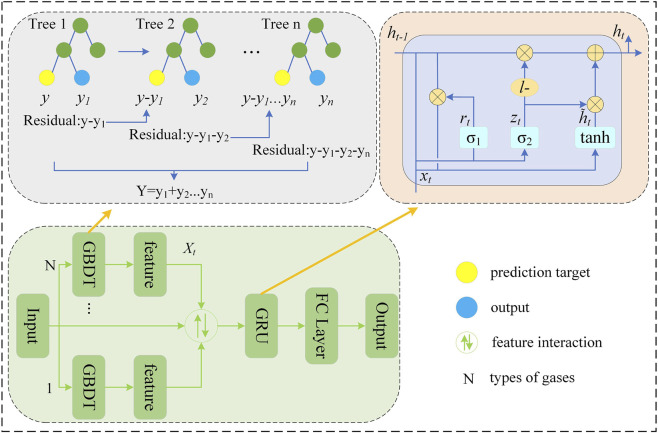
JGPM network architecture diagram.

Traditional gas concentration prediction models involve complex manual feature preprocessing, which increases the complexity of the prediction process and the uncertainty of the prediction results (Peng et al., 2023). This paper proposes a novel GBDT-GRU Joint Prediction Model (JGPM) to address this issue. The model architecture is shown in Figure 6. This network architecture combines the GBDT and GRU models. First, the GBDT model is responsible for extracting complex nonlinear features from the one-dimensional time series data collected by the sensors, performing preliminary predictions for the N target variables (where N represents the number of gas types), and outputting the prediction results. Next, the prediction results from the GBDT model are subjected to feature interaction with the original data and then refined further using the GRU model. The GRU captures the dynamic changes in the time series through its gating mechanism, allowing for better modeling of the temporal evolution of gas concentrations. The overall architecture enhances the model’s performance in gas concentration prediction tasks by combining nonlinear feature extraction with sequence modeling. JGPM uses GBDT for preliminary prediction of the sensor data, avoiding the need for complex manual feature extraction. At the same time, JGPM, through multi-target modeling, shared features, and a multi-output structure, is capable of simultaneously predicting the concentrations of different types of gases.

## 4 Results and discussion

To ensure consistency in the experiments, all algorithms were implemented in a Python 3.10 environment using PyCharm Community Edition 2022.3.3. The hardware configuration of the computer is as follows: AMD Ryzen 5 7500F CPU, NVIDIA GeForce RTX 4060 (8 GB VRAM) GPU, and 16 GB RAM. For the gas classification and concentration prediction tasks, 1920 GASF images and 1920 time-series sampling sequences were constructed for model training and evaluation, respectively. To ensure the reliability and robustness of the evaluation, five-fold cross-validation was adopted for the gas classification task. During training, a weight decay strategy was applied with a decay coefficient of 1 × 10^−4^. In addition, to ensure stable convergence, a cosine annealing learning rate scheduler was employed, with an initial learning rate of 0.0004 gradually decaying to a minimum of 1 × 10^−6^ over the training process. For the gas concentration prediction task, considering the strong temporal correlation among samples within the same gas exposure cycle, group-based cross-validation was adopted to prevent data leakage and ensure strict independence between the training and testing sets.

### 4.1 Evaluation metrics

For the qualitative gas identification and quantitative gas detection tasks, four and three evaluation metrics were selected, respectively, to assess the predictive performance of the proposed models. Table 3 presents the calculation methods for these seven metrics. Furthermore, to comprehensively evaluate the efficiency of the classification models, four additional metrics were introduced: Floating Point Operations (FLOPs) for measuring computational complexity, model size (Size) for indicating storage overhead, inference latency (Latency) for quantifying the processing time of a single input sample, and the number of parameters (Params) for representing the total scale of trainable parameters.

**TABLE 3:** Evaluation metrics for two tasks.

Task	Name	Formula
Gas qualitative identification	Accuracy	$\text{ACC}=\frac{\text{TP}+\text{TN}}{\text{TP}+\text{FP}+\text{FN}+\text{TN}}$
	Precision	$\text{Precision}=\frac{\text{TP}}{\text{TP}+\text{FP}}$
	Recall	$\text{Recall}=\frac{\text{TP}}{\text{TP}+\text{FN}}$
	Cohen’s Kappa	$K=\frac{P_{o}-P_{e}}{1-P_{e}}$
Gas quantitative detection	MSE	$MSE=\frac{1}{n}\sum_{i=1}^{n}{\left(y_{i}-{y_{i}}^{*}\right)}^{2}$
	RMSE	$RMSE=\sqrt{\frac{1}{n}\sum_{i=1}^{n}{\left(y_{i}-{y_{i}}^{*}\right)}^{2}}$
	*R* ^2^	$R^{2}=1-\frac{SSE}{SST}=1-\frac{{\sum_{i=1}^{n}\left(y_{i}-{y_{i}}^{*}\right)}^{2}/n}{{\sum_{i=1}^{n}\left(y_{i}-{\overset{-}{y}}_{i}\right)}^{2}/n}$

### 4.2 Classification

**FIGURE 7:**
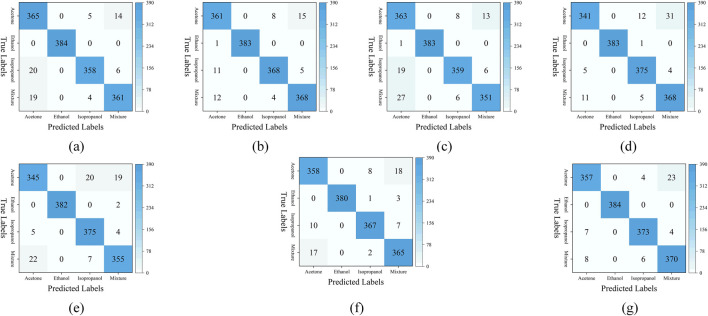
Confusion matrices for the classification task, where **(a–g)** correspond to the confusion matrices of AlexNet, DenseNet, MobileNetV3, ResNet50, ShuffleNet, PSCFormer, and LLGFN on the mixed gas dataset, respectively.

As shown in Table 4, a comprehensive evaluation was conducted on the overall performance of LLGFN, PSCFormer, and five mainstream baseline models in terms of classification accuracy and lightweight metrics. Benefiting from the deep integration of DW convolution-based downsampling, the Transformer encoder, and the LFE module, LLGFN significantly outperformed the other algorithms in classification performance, achieving the highest average accuracy of 96.20% under 5-fold cross-validation. Compared with classical networks known for their high accuracy, LLGFN further surpassed DenseNet (95.98%) and ResNet50 (95.29%) in classification accuracy, demonstrating superior feature extraction capability. This significant performance advantage can be attributed to the customized architectural design of LLGFN tailored to the specific data modality. Unlike natural images with typical spatial topological structures, images generated from one-dimensional gas sensor data through GASF transformation highly condense the inter-sensor correlations, where slight variations in gas concentration or gas species are often manifested only as subtle perturbations in local textures. Therefore, while capturing the global distribution, the model must also pay particular attention to fine-grained local features. Traditional networks such as ResNet50 and DenseNet are primarily designed for global semantic extraction in natural images and thus tend to overlook critical local features in GASF images. Furthermore, the confusion matrix analysis shown in Figure 7 indicates that conventional models are prone to misclassifying acetone due to the highly similar response intensities of isopropanol, mixed gases, and acetone. LLGFN adopts a parallel global–local dual-branch architecture, where the LF module utilizes progressively expanded channels and large convolution kernels to extract global features from GASF images, while the GF module employs a multi-head self-attention mechanism to precisely capture fine-grained local information. This mechanism effectively enhances the model’s discriminative capability for highly similar gases and substantially reduces the misclassification rate. In contrast, AlexNet, MobileNetV3, and ShuffleNet exhibit relatively inferior classification performance. The limitation of AlexNet lies in its reliance on large convolution kernels and massive fully connected layers, while lacking residual or dense connection mechanisms, which makes it prone to overfitting in the current task and consequently limits its generalization capability. In contrast, the accuracy limitations of MobileNetV3 and ShuffleNet arise from the excessive pursuit of computational cost reduction at the expense of the models’ representation capability. LLGFN not only overcomes the aforementioned limitations but also demonstrates significant advantages in lightweight metrics. Compared with ResNet50 and AlexNet, LLGFN contains only 1.59M parameters (Params) and a model size (Size) of merely 6.98 MB, achieving an order-of-magnitude reduction in storage overhead. While maintaining an extremely low computational complexity comparable to lightweight networks specifically designed for mobile devices, such as MobileNetV3 and ShuffleNet (with FLOPs of only 0.20G), LLGFN improved the classification accuracy by 1.58% and 1.92%, respectively, achieving an optimal balance between performance and computational cost. Although PSCFormer demonstrates exceptional lightweight potential, with FLOPs of only 0.02G and a model size of 1.18 MB, LLGFN achieves a decisively higher classification accuracy (96.20% vs. 95.60%) for gas classification tasks with extremely low fault tolerance, at the cost of only a marginal increase in model size. Moreover, LLGFN exhibits a slightly lower inference latency than PSCFormer (4.84 ms vs. 4.94 ms). These results fully demonstrate that LLGFN achieves a superior trade-off between high accuracy and lightweight design.

**TABLE 4:** Classification results.

Models	Avg. Accuracy	FLOPs (G)	Size (MB)	Latency (ms)	Params
AlexNet	94.83%	1.42	217.51	4.85	57020228
DenseNet	95.98%	5.79	27.13	20.47	6957956
MobileNetV3	94.62%	0.13	6.48	4.79	1664508
ResNet50	95.29%	8.26	90.02	5.06	23516228
ShuffleNet	94.28%	0.27	3.61	5.28	901492
PSCFormer	95.60%	0.02	1.18	4.94	300742
LLGFN	96.20%	0.20	6.98	4.84	1593056

**FIGURE 8:**
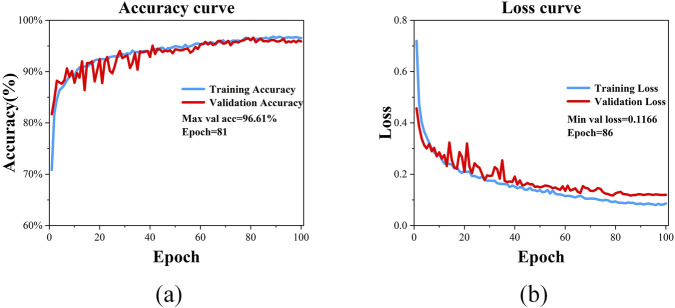
Learning curves: **(a)** accuracy curve; **(b)** loss curve.

In terms of inference efficiency, which is critical for practical applications, the single-image inference latency of LLGFN is only 4.84 ms, which is substantially lower than that of DenseNet (20.47 ms) and slightly better than that of ResNet50 (5.06 ms). This extremely low latency demonstrates that the proposed model can achieve rapid response without relying on complex computational resources, thereby fully satisfying the stringent requirements of real-time detection tasks on edge computing devices. Finally, Figure 8 illustrates the learning curves of one fold during the 5-fold cross-validation process. As shown in the figure, the training and validation curves converge smoothly and closely align with each other, without exhibiting obvious overfitting or underfitting phenomena, further demonstrating the excellent training stability and generalization capability of LLGFN.

### 4.3 Ablation experiment

To systematically evaluate the effectiveness of each component in the proposed LLGFN architecture, this paper conducts comprehensive ablation experiments. By selectively removing or retaining the CNN branch, Transformer branch, and the proposed LFE, the contribution of each module to the overall model performance is analyzed. All ablation experiments were conducted under the same experimental configuration and evaluation criteria to ensure the comparability and reliability of the results. Specifically, LLGFN-A represents the basic CNN structure without the Transformer branch and LFE, LLGFN-B represents the CNN-Transformer parallel structure with the LFE removed, and LLGFN-C represents the CNN structure with only the LFE introduced, excluding the Transformer branch.

The experimental results, as shown in Table 5, indicate that when using the basic CNN as the feature extraction network, the model achieved an accuracy of 93.82% and a Cohen’s Kappa of 91.75%. The results indicate that CNN is effective in capturing the local spatial structural features in gas images formed by the Gramian matrix, such as the correlation patterns between adjacent sensor responses and the local texture information after color mapping. However, gas images inherently encode the global response relationships of the sensor array at the same sampling moment. Relying solely on convolution operations makes it difficult to fully model the long-range correlations between different sensor channels, thus limiting further improvement in overall discriminative performance. When only the LFE is removed, the model performance improves significantly, with an accuracy of 95.38% and a Cohen’s Kappa of 93.84%. This indicates that effective cross-branch feature fusion has been achieved for gas images constructed from sensor array responses. When only the LFE is included in the CNN, the accuracy drops to 94.40% and Cohen’s Kappa to 92.53%, but still higher than the basic CNN. This demonstrates the effectiveness of the LFE.

**TABLE 5:** Ablation experiment results.

Models	Highest accuracy	Precision	Recall	Cohen’s Kappa
LLGFN-A	93.82%	93.95%	93.82%	91.75%
LLGFN-B	95.38%	95.54%	95.38%	93.84%
LLGFN-C	94.40%	94.45%	94.40%	92.53%
LLGFN	96.61%	96.64%	96.61%	95.49%

As shown in Table 5, the CNN branch, Transformer branch, and LFE each play complementary and irreplaceable roles in the gas image classification task. The CNN branch focuses on extracting local spatial structures and texture features from the gas images, while the Transformer branch effectively models the global response relationships across sensors. The LFE, on the other hand, plays a role in multi-scale feature extraction and lightweight processing. The complete model achieves optimal performance across all evaluation metrics, fully validating the rationality and effectiveness of the three modules proposed in LLGFN for gas sensor array image feature modeling.

### 4.4 Single gas concentration prediction

To evaluate the performance of JGPM in the concentration prediction task, comparative analyses were conducted against two baseline models, namely, GBDT and GRU. As shown in Table 6, JGPM achieved the best prediction performance across all gas categories and evaluation metrics, significantly outperforming both GBDT and GRU. Specifically, the GBDT model exhibited the highest prediction errors across all tasks. In particular, for acetone concentration prediction, its mean squared error (MSE) reached 0.9624, indicating that shallow learning methods are unable to effectively model complex nonlinear concentration variations. Although the GRU model improved temporal feature extraction through the introduction of a gated recurrent mechanism, resulting in reduced error metrics, its prediction results still exhibited relatively high variance, indicating insufficient stability. In contrast, the proposed JGPM model achieved a substantial reduction in prediction errors. Particularly for isopropanol concentration prediction, the MSE and RMSE of JGPM were reduced to 0.2087 and 0.4563, respectively, while the *R* ^2^ reached as high as 0.9899, indicating an exceptionally accurate linear fit between the predicted values and ground-truth concentrations.

**TABLE 6:** Single gas concentration prediction results.

Models	Gas types	MSE	RMSE	*R* ^2^
GBDT	Acetone	0.9624 ± 00934	0.9798 ± 0.0475	0.9537 ± 0.0059
	Ethanol	0.8107 ± 0.1857	0.8946 ± 0.1016	0.9607 ± 0.0113
	Isopropanol	0.4595 ± 0.0493	0.6769 ± 0.0357	0.9778 ± 0.0038
GRU	Acetone	0.6160 ± 0.2340	0.7712 ± 0.1458	0.9705 ± 0.0110
	Ethanol	0.7070 ± 0.3195	0.8196 ± 0.1876	0.9668 ± 0.0138
	Isopropanol	0.4117 ± 0.1315	0.6332 ± 0.1039	0.9800 ± 0.0072
JGPM	Acetone	0.4359 ± 0.0513	0.6591 ± 0.0378	0.9789 ± 0.0040
	Ethanol	0.3932 ± 0.1161	0.6197 ± 0.0956	0.9810 ± 0.0065
	Isopropanol	0.2087 ± 0.0190	0.4563 ± 0.0206	0.9899 ± 0.0016

Furthermore, the standard deviations of JGPM across all evaluation metrics were significantly lower than those of GBDT and GRU. These low numerical fluctuations strongly demonstrate the superior prediction stability and robust anti-interference capability of JGPM. The results indicate that the feature interaction mechanism of JGPM is capable of establishing a highly robust mapping relationship between feature sequences and gas concentrations.

### 4.5 Mixed gas concentration prediction

Table 7 presents the concentration prediction results of acetone, ethanol, and isopropanol in mixed gases using JGPM. Due to the cross-sensitivity characteristics of gas sensors, concentration prediction in mixed gases is more challenging than that in single-gas scenarios. As shown in Table 7, the GBDT model achieved high *R* ^2^ values across all three gases, indicating that the gradient boosting tree–based nonlinear regression method can effectively capture the static nonlinear mapping relationship between sensor array responses and gas concentrations. However, its MSE and RMSE remain relatively high, indicating that GBDT has limitations in modeling complex temporal dynamics of mixed gases. Although GRU can explicitly model the dynamic dependencies of time series, its MSE and RMSE for all three gases are slightly higher than those of GBDT, while *R* ^2^ also shows a slight decrease. This suggests that end-to-end recurrent modeling alone is insufficient to fully capture the deep nonlinear feature structures embedded in complex mixed-gas data. In contrast, after introducing JGPM, the model achieved the best performance in the mixed-gas concentration prediction task. Taking isopropanol as an example, the MSE and RMSE of JGPM were reduced to 0.1768 and 0.4177, respectively, while *R* ^2^ increased to 0.9914. Furthermore, JGPM exhibits the lowest standard deviation across all evaluation metrics, indicating that it can effectively integrate the advantages of GBDT in nonlinear feature modeling and GRU in temporal dynamic modeling. As a result, it significantly improves both the accuracy and stability of mixed-gas concentration prediction.

**TABLE 7:** Concentration prediction results of acetone, ethanol, and isopropanol in mixed gases.

Models	Gas types	MSE	RMSE	*R* ^2^
GBDT	Acetone	0.3725 ± 0.0652	0.6080 ± 0.0530	0.9820 ± 0.0042
	Ethanol	0.3564 ± 0.0567	0.5952 ± 0.0463	0.9827 ± 0.0039
	Isopropanol	0.3749 ± 0.0754	0.6093 ± 0.0611	0.9818 ± 0.0047
GRU	Acetone	0.3757 ± 0.0642	0.6108 ± 0.0512	0.9818 ± 0.0042
	Ethanol	0.3707 ± 0.0500	0.6075 ± 0.0408	0.9821 ± 0.0033
	Isopropanol	0.3779 ± 0.0610	0.6128 ± 0.0484	0.9817 ± 0.0041
JGPM	Acetone	0.1819 ± 0.0465	0.4232 ± 0.0533	0.9912 ± 0.0028
	Ethanol	0.1827 ± 0.0459	0.4240 ± 0.0539	0.9911 ± 0.0027
	Isopropanol	0.1768 ± 0.0405	0.4177 ± 0.0477	0.9914 ± 0.0025

**FIGURE 9:**
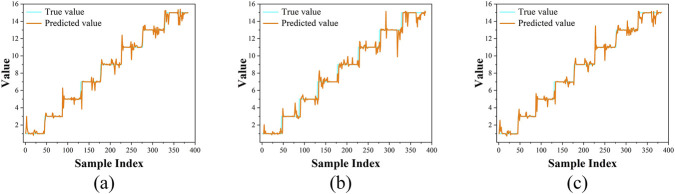
Fitting plots of the true and predicted concentrations of each component in the mixed gas, where **(a–c)** represent acetone, ethanol, and isopropanol, respectively.

To more intuitively illustrate the fitting dynamics of the JGPM model in quantitative detection of mixed gases, Figure 9 presents the comparison curves between the true and predicted concentrations of three gas components-acetone (a), ethanol (b), and isopropanol (c)-as functions of the sample index. As shown in Figure 9, under the experimentally designed stepwise concentration variations, the predictions of the JGPM model closely follow the trends of the true concentrations. In particular, in the step-transition regions where abrupt concentration changes occur, the model exhibits strong dynamic response capability, rapidly tracking the new concentration levels with negligible response delay. In the steady-state regions where the concentrations remain constant, although the sensor signals contain inherent background noise, the model predictions maintain high stability, exhibiting only minor random fluctuations without noticeable systematic deviation or baseline drift. These results are highly consistent with the quantitative evaluation metrics reported in Table 7, further providing intuitive evidence that the proposed JGPM model achieves excellent prediction accuracy and strong robustness in complex mixed-gas detection tasks.

## 5 Conclusion

This paper proposes a lightweight Local–Global Feature Fusion Network (LLGFN) and a GBDT–GRU Joint Prediction Model (JGPM), aiming to address the limitations of traditional CNNs and Transformers in balancing local and global feature modeling, their large parameter sizes, and their reliance on manual feature extraction. LLGFN adopts an LF–GF parallel architecture, in which the LF module enhances local feature representation through cross-stage feature connections and the lightweight, efficient LFE, while the GF module performs global context modeling using a downsampled Transformer encoder reconstructed with depthwise convolutions. Finally, global and local features are effectively fused via the FI module. LLGFN achieved an average classification accuracy of 96.20% in the gas classification task while significantly reducing the number of model parameters, demonstrating its lightweight design. Ablation experiments further verify the effectiveness of each module. JGPM combines the nonlinear feature extraction capability of GBDT with the temporal dynamic modeling ability of GRU to achieve high-precision prediction of gas concentration evolution over time, and demonstrates higher accuracy and robustness compared with single GBDT and GRU models in comparative experiments. However, breathomics in clinical scenarios involves highly complex mixtures of VOCs. This study, which focuses on the analysis of only three key types of markers, possesses certain limitations (Binson et al., 2025; Khanna et al., 2025; Binson et al., 2024a). Future work will further integrate real-time clinical data and incorporate cutting-edge research findings on multi-component sensor arrays and ensemble machine learning to continuously enhance the system’s robustness and diagnostic precision in complex clinical environments. Future research will be dedicated to integrating perovskite quantum dot materials into the sensor array. By leveraging their exceptional detection sensitivity and composition-tunable selectivity (Zhang et al., 2025; Fang et al., 2025), we aim to fundamentally optimize the sensing evolution characteristics of chemical sensors. This approach is intended to effectively address the identification challenges posed by multi-component VOC mixtures in complex clinical environments.

### 5.1 Resource identification initiative

Catalog numbers (and RRIDs where available) for key resources (e.g., the CGS-8 gas sensing system, sensor types in the array, and major software/tools used for analysis) are provided in the Methods and Table 1. For items without RRIDs, manufacturer and model information are reported.

## Data Availability

The raw data supporting the conclusions of this article will be made available by the authors, without undue reservation.
